# Social Marketing and Breastfeeding: A Literature Review

**DOI:** 10.5539/gjhs.v5n3p82

**Published:** 2013-02-08

**Authors:** Manuela Schmidt

**Affiliations:** 1 School of Health and Society, Kristianstad University, Kristianstad, Sweden

**Keywords:** social marketing, breastfeeding, literature review, education, psychology, behavioural sciences

## Abstract

**Aims:**

Through the review of relevant literature this study illuminates the concepts of social marketing and breastfeeding. It specifically discusses the positioning of the link between social marketing and breastfeeding within different fields of study and develops a theoretical framework that tries to bridge the gap between those disciplines.

**Method:**

Various electronic databases were used and through systematic selection 11 scientific articles were identified that this literature review is based on.

**Results:**

The review indicates that the relationship between social marketing and breastfeeding is complex. There are indications that this relationship is being investigated within three distinct fields of research: psychology/education, public health and marketing. Depending on the research field the emphasis is put on either breastfeeding or social marketing as well as on the other concepts that were discovered to be of importance within this relationship. Namely, group and individual demography as well as behaviour were revealed to be important elements of the link between social marketing and breastfeeding.

**Conclusions:**

Based on the results this study concludes that a more multidimensional view on the relationship between the concepts under study is needed since the focus of previous studies is very one-sided and limited to just one element when all elements should be integrated equally.

## 1. Introduction

Health promotion work aims at enabling and empowering an individual, a community or an organization to find the strength and control to take action to improve their life situation and their health (WHO, 1986, 2005, 2009). Social marketing (SM) has currently emerged as a popular tool in health promotion, being given special attention in recent public health literature. This popularity is based on reasonable evidence that carefully managed SM programmes can be very effective (Andreasen, 2006; Bryant, 2010; Grier & Bryant, 2005; Nutbeam, Harris, & Wise, 2010; Stead, Gordon, Angus, & McDermott, 2007). An increasing number of studies started thus to inquire into the relationship between SM and specific public health concerns such as tobacco use (e.g. Pechmann & Reibling, 2000; Wakefield, Flay, Nichter, & Giovino, 2003), HIV/AIDS communication (e.g. Bertrand, O’Reilly, Denison, Anhang, & Sweat, 2006; Myhre & Flora, 2000), or drinking and driving (e.g. Elder et al., 2004), among others. Promoting breastfeeding (BF) has been a current matter of public health agencies and different health organizations throughout the last decades ((US) Department of Health and Human Services, 2000, 2011; Fairbank et al., 2000; Morrow et al., 1999; Raj & Plichta, 1998; WHO/UNICEF, 1990) and SM has proven to be a very helpful and powerful tool in this context (Centers for Disease Control and Prevention, 2005; Manoff, 1985; Stead, Hastings, & McDermott, 2007). However, many public health professionals still have an incomplete understanding of SM (Hill, 2001; McDermott, 2000) that is dominated by the criticism that SM focuses too much on individual behaviour rather than on the underlying environmental and social causes of the problem it addresses (Donovan & Henley, 2003; Grier & Bryant, 2005).

### 1.1 Social Marketing

The field of health communication has been rapidly changing over the past two decades. Once working with a one-dimensional reliance on public service announcements it has now evolved to a more sophisticated approach which draws from successful techniques used by commercial marketers, termed ‘social marketing’. Rather than dictating the way that information is to be conveyed from the top-down, public health professionals are learning to listen to the needs and desires of the target audience themselves, and constructing the programme from there (Weinreich, 2006). From a health promotion point of view SM is accepted as a model for communication to bring about behaviour change (Nutbeam et al., 2010) though it should be viewed more as a framework or structure that draws from many other bodies of knowledge such as psychology, sociology, anthropology and communication theory to understand how to influence people’s behaviour (P. Kotler & Zaltman, 1971). It is said that a question raised by Wiebe in 1952 “*Why can’t we sell brotherhood like you sell soap?*” gave birth to SM as a field (P. Kotler & Zaltman, 1971; Wiebe, 1952).

SM, when compared with commercial marketing, (CM) has many aspects in common. It was Kotler, the most established researcher in the field of marketing, who developed the concept of SM further. He defines SM as

*“…the use of marketing principles and techniques to influence a target audience to voluntarily accept, reject, modify, or abandon a behaviour for the benefit of individuals, groups, or society as a whole… [it] is largely a mix of economic, communication, and educational strategies… As a last resort, the social marketer may turn to the law or courts to require a certain behaviour”* (Philip Kotler, Roberto, & Lee, 2002, pg. 19-20).

The major difference between SM and CM is whereas CM’s main aim is profit-oriented (it should benefit the marketer); the objective of SM is to benefit the target audience, thus the welfare of an individual, a group or society in general (Philip Kotler et al., 2002). Both marketing forms, however, have many aspects in common and try to change people’s behaviour as well as focus primarily on the consumer -on learning what people want and need (Green & Tones, 2010; Philip Kotler et al., 2002; Nutbeam et al., 2010; Weinreich, 2006). The planning process takes this consumer focus into account by addressing the elements of the marketing mix. This refers to decisions about the conception of a Product, Price, distribution (Place), and Promotion, also called the ‘Four Ps’ of marketing (McCarthy, 1960). SM complements with additional ‘P’s.’, such as Public, Partnership, Policy and Purse strings (Weinreich, 2006).

The last few years have witnessed a growth in interest in SM, both in research and practice, by policy makers, practitioners, and health professionals (Gordon, 2006). SM has proven to change behaviour significantly for the sake of improving health (e.g. quitting smoking), improving safety and preventing injuries (e.g. use of bicycle helmets, water wings), protecting the environment (e.g. water quality, use of fertilizers) or contributing to the community (e.g. voting) (Philip Kotler et al., 2002). The majority of studies show positive effects of the use of SM in health promotion, however some authors have recently raised concerns that the message of SM might be misunderstood and either have none or negative effects depending on implementation. Moreover there might even be possibilities of boomerang effects of SM that instead of discouraging certain behaviour it creates awareness of that behaviour (Hastings, Angus, & Bryant, 2011). This literature study will exclusively focus on SM in the context of improving health, in particular the health of infants through proper nutrition, namely BF.

### 1.2 Breastfeeding

Malnutrition is responsible, directly or indirectly, for about one third of deaths among children under five worldwide (WHO, 2012). Above two thirds of these deaths, often associated with inappropriate feeding practices, occur during the first year of life. Thus nutrition and nurturing during the first years of life are both crucial for life-long health and well-being. World Health Organization (WHO) recommends that infants start breastfeeding within one hour of life, are exclusively breastfed for six months, with the timely introduction of adequate, safe and properly-fed complementary foods while continuing breastfeeding for up to two years of age or beyond (WHO, 2011).

Empirical studies in the field have provided evidence that BF is associated with health benefits in infants (Campbell & Jones, 1996). BF was found to be associated with a decreased risk for gastrointestinal diseases and infectious illnesses in the first year of life (e.g. Aniansson et al., 1994; Cunningham, 1979; Howie, Forsyth, Ogston, Clark, & Florey, 1990; Kramer et al., 2001; Wang & Wu, 1996) as well as a decrease in necrotizing enterocolitis and neonatal infections (Narayanan, Prakash, Murthy, & Gujral, 1984). Further benefits of BF were found in low birth weight babies (Meier, Engstrom, Mingolelli, Miracle, & Kiesling, 2004). While the number of studies and their results could be considered to be robust, the interpretation of aforementioned and similar studies should still be interpreted with caution

*“…as adjustment for known risk factors cannot control for as yet unrecognized confounders or differences in parenting associated with breastfeeding”* (Campbell & Jones, 1996, pg. 408).

Moreover the context could yet be another factor that should be considered (Campbell & Jones, 1996). Acknowledging the potential moderators in the relationship between BF and infant health outcomes, this article puts emphasis on the promotion of BF through the use of SM (being one of health promotion tools), rather than trying to contribute to the investigation of the moderators.

### 1.3 Aims

Even though SM has a relatively short history in encouraging behaviour change (Quinn, 2010) it has already proven to be a powerful tool in health promotion work. Several studies have been published, evidencing that SM provides a promising framework to address health promotion aspects (e.g. Do Paço, 2010; Gordon, 2006; Huhman et al., 2005; Lefebvre & Flora, 1988; Ling, 1992; Maddock et al., 2007; Oglethorpe, 1995; Rothschild, 1999; Walsh, 1993). While most literature reviews focus either on the concept of SM or BF, this study will concentrate on the relationship between both of them and how it is viewed in different fields of research. By doing so this paper aims at constructing a theoretical framework that could be applied and used for future empirical studies that investigate the relationship between SM and BF. Particular focus in developing this framework is put on the need of bridging the gap between different disciplines within which the link between the two concepts is being investigated, in order to illuminate the potential benefits of a multi-disciplinary approach to the relationship between SM and BF.

## 2. Method

For the purpose of a comprehensive review of contemporary research on SM together with BF, a systematic literature search was conducted in a similar style to previously published literature reviews (Jensen, 2011; Söderlund, Fischer, & Johansson, 2009; Weed et al., 2012). For a more efficient search large electronic databases were used that combined different research disciplines. Searches were conducted on Lund University’s former database LIBHUB (that included Cochrane, UpToDate, Best Practice and MD Consult with primary focus on clinical and evidence-based research) as well as on PubMed (that includes biomedical literature from MEDLINE and life science journals), Ebscohost and PsycINFO. The search included articles published over a time period of the last 16 years (1996-2011). A key word search was conducted including the following key words: ‘social marketing’ AND breastfeeding (OR breast feeding OR breast-feeding) resulting in a total of 139 articles. After excluding a small number of articles written in a language other than English and a substantial amount of duplicates generated by the different databases, all abstracts were read by the author. This formed the basis for the selection of publications to be studied more in detail. Articles were excluded from the sample if e.g. BF and/or SM were used as tangible examples rather than as the primary focus of the studies. The final review consisted of 11 articles which were considered to fit the purpose of this study.

### 2.1 Method Discussion

By using SM as a key term the author of this paper was guided by the idea that researchers using the term have been doing so in full awareness of its inherited differences from narrowly defined advertising campaigns and/or health promotion campaigns, which several practitioners sometimes confuse with SM (Stead & Hastings, 1997). This might give uprise to the possibility that articles that actually involved SM as a concept were missed by the selection process which should be put forward as a possible limitation of the review performed.

A geographical limitation was not considered as relevant since it would not have resulted in a satisfying amount of research literature for the purpose of this paper though it was acknowledged that a geographical/ethnical/cultural focus could be of interest in future studies that focus on either BF e.g. BF among Hispanics or SM e.g. use of SM technique in Europe. The articles included in this study refer mostly to SM campaigns taken up in the UK or, with a few exceptions, in the US, but campaigns from other regions were not consciously excluded from the search. Only empirical studies, i.e. those including a data collection, were of interest in order to have a stronger focus on the outcome. Theoretical papers were therefore excluded.

## 3. Result

Scientific articles found on the topic of SM and BF are summarized in Table 1. Firstly all the articles found have a common theme that serves as a core for all the studies, namely the idea of SM as a tool for BF promotion, which resonates well with the problem in hand. Further the studies reviewed can be subdivided into disciplines in order to indicate which disciplinary tradition the articles used as well as to indicate the discipline to which the studies under review make their contribution. Thus the result presentation that follows is based on the disciplinary division of the papers in order to present a coherent picture along the disciplinary lines, which will then be reflected on through a broader prism in the discussion section.

**Table 1:** Summary of the articles included in the review

Author(s)	Year	Title	Discipline	Level(s) of analysis
Whitelaw S, Smart, E, Kopela, J, et al.	2011	Developing social marketing capacity to address health issues	Education	Group/Social
Brown CA, Poag S, Kasprzycki C	2001	Exploring large employers’ and small employers’ knowledge, attitudes, and practices on breastfeeding support in the workplace	Psychology	Group/Organizational
Wright AL, Naylor, A, Wester, R, et al.	1997	Using Cultural Knowledge in Health Promotion: Breastfeeding among the Navajo	Education & Psychology	Group/Social
Mitra, AK, Khoury, AJ, Hinton, AW, et al.	2004	Predictors of Breastfeeding Intention Among Low-Income Women	Public Health	Individual/Group/Social
Sherriff, N, Hall, V	2011	Engaging and supporting fathers to promote breastfeeding: a new role for Health Visitors?	Public Health	Individual/Group/Social
Lowry, RJ, Billett, A, Buchanan, C, et al.	2009	Increasing breastfeeding and reducing smoking in pregnancy: a social marketing success improving life chances for children	Public Health	Individual/Group/Social
Lindenberger, JH, Bryant, CA	2000	Promoting breastfeeding in the WIC Program: a social marketing case study	Public Health	Individual/Group/Social
Labbok, MH, Wardlaw, T, Blanc, A, et al.	2006	Trends in exclusive breastfeeding: findings from the 1990s	Public Health	Social
Fridinger, F, Alfonso, ML, Hussain, A, et al.	2003	A multi-year profile of Public beliefs and attitudes regarding breastfeeding practices	Public Health	Individual/Group/Social
Mattson, M, Basu, A	2010	The message development tool: a case for effective operationalization of messaging in social marketing practice	Marketing	Group/Organizational
Lowry, R, Austin, J, Patterson, M	2011	Using Social Marketing to Improve Breast-Feeding Rates in a Low Socioeconomic Area	Marketing	Group/Organizational

### 3.1 Psychology and Education

Three studies have been identified that investigated the relationship between SM and BF within the broadly defined field of psychology and education.

A study by Whitelaw, Smart, Kopela, Gibson, and King (2011) focuses on the implementation of an educational programme, with its base in SM techniques, to achieve a wider understanding and practice of BF among young people. The paper is based on a case study grounded on multiple sources of data collected through interviews, focus groups, stakeholder workshops, and surveys among others, and performed within the frame of the National Health Service board initiative in rural Scotland. The case study was conducted with an action research method, where the authors of the paper were not only passive observers but also part of the action team designed to promote BF practices among young people. The main outcome of the study was that through the use of SM techniques, primarily through information dissemination and pedagogical activities, the understanding of the importance of BF among health care professionals and young people (groups that were targeted in the project) would increase, as well as further dissemination of knowledge to young people in rural Scottish areas would be achieved. Another important outcome reported in the paper was policy implications namely the identification of the core issues for the sustainability of BF practices in the rural population. Long-term planning mechanisms, managerial support on the local government level as well as security of on-going resources were deemed vital for the success of SM activities related to BF promotion among young adults. The paper spans two levels of analysis: group level represented by young people as well as societal level with the discussion of an overall policy of BF.

The study by Brown, Poag, and Kasprzycki (2001) takes a more psychological view on the link between SM and BF. The article investigates how employers’ knowledge, attitude and practices in BF support to employees can be served as an SM tool in BF promotion. Data were collected by means of focus groups divided into employers and employees. Through triangulation techniques (mixed method) the researchers have found important categories that interlock employers’ and employees’ understanding of BF practices. Those categories included were ‘knowledge and attitude’, ‘practices and experiences of BF in the workplace’, ‘BF as employee-wellness’, ‘barriers to providing BF support’, ‘motivators for providing BF support’, and ‘communication and marketing strategies’. Overall, the identification of these categories has pinpointed themes for future research aiming at investigating empirically the employee-employer relationship in BF practices and the role of the employer as an SM ‘actor’. Moreover the study has identified important practice implications in relationship to BF practice promotion at the workplace. The study spans two levels of analysis: on the group level it targets the employee vs. employer relationship and thus presents a complex inter-group interaction; the study also addresses the organizational level of analysis by elevating the analysis to business policy level.

The third article in this subsection by Wright, Naylor, Wester, Bauer, and Sutcliffe (1997) is positioned between the fields of psychology and education and thus illuminates the variety of ideas presented in the articles by Whitelaw et al. (2011) and Brown et al. (2001) presented above. The paper deals with SM and community participation techniques in the promotion of BF in the Navajo Indian reservation in the US. The aim of the study was to describe how SM could promote and institutionalize BF among Native Americans. The research has used both qualitative and quantitative techniques for data collection, which allowed for rich and complex data to be available for the analysis. The results of the study indicate that defining the problem of non-breastfeeding, illuminating the risk involved, collaboration with local institutions and individuals, reinforcements of BF practices as well as overall institutional change in the health care system could lead to changes of behavioural attitudes and the embracement of BF practices. The study spans two levels of analysis: the group level is represented by the concentration on women in one particular ethnic group, and the societal level is represented by the investigation of community practices and influences on BF behaviour.

### 3.2 Public Health

Articles reviewed within the field of public health are generally based on the idea that SM is viewed as a reinforcement of women’s perception and practices of BF streaming from their social, cultural, socio-economic and psychological characteristics (Fridinger et al., 2003; Labbok, Wardlaw, Blanc, Clark, & Terreri, 2006; Lindenberger & Bryant, 2000; R. J. Lowry, Billett, Buchanan, & Whiston, 2009; Mitra, Khoury, Hinton, & Carothers, 2004; Sherriff & Hall, 2011).

Lindenberger and Bryant (2000) as well as Mitra et al. (2004) have directed their attention to the view and practices of BF among women with a low income, who have been identified as having the lowest BF rates in comparison to other income-level groups. Through investigating this group of women the researchers have observed that using SM programmes that include provision of knowledge of the benefits associated with BF, as well as some type of social support from the health professionals, has a positive effect on BF practices among low-income women.

Fridinger et al. (2003) have addressed the racial and ethnic group perceptions of BF and SM influences. As in the paper by Lindenberger and Bryant (2000) and Mitra et al. (2004), Fridinger et al. (2003) address the issue of social support as well, not only from the public health care professionals’ view but also from the immediate social network view (e.g. family). It is this support that is considered to be of great importance for BF practice. In line with previous research the paper identifies income as a strong predictor of BF at the same time highlighting ethnicity as an important element influencing behaviour related to BF. Based on the conclusions of the survey performed within the paper the authors argue that SM targeting strategies are important in the process of delivering knowledge and understanding of BF practices to specific demographic groups of women.

Sherriff and Hall (2011) take a more unorthodox view of BF practices. Instead of concentrating on the SM programmes they consider the individual who can be used as an SM tool – namely the father. While still looking at the socio-economic groups and motivating the selection criteria by social class, the authors of the paper have examined the father’s role in promoting BF activities. The results of the study indicate that fathers’ attitudes towards BF are an important factor in motivating and encouraging women to breastfeed, which reinforces the ideas in the articles presented above that social support -in this case the spouse’s support- is an important moderator and SM tool in BF practices.

Lowry et al. (2009), in contrast to other studies in the subsection, have taken a broader social approach to BF practices among women in one specific community in the UK. The emphasis in the article has been put on SM as a tool in forming women’s opinions on smoking and BF before and after child birth. The SM tool developed by the researchers has shown to be effective in smoking reduction and increase in BF practices among the local women.

Lastly, the article by Labbok et al. (2006) broadens the discussion on SM and BF by offering the idea of SM as a trend and BF as being influenced by this trend. The article, as all other articles in this section, has demographic characteristics such as culture and socio-economic factors as a starting point, while focusing on the interventive trend-setting capacity of the baby-friendly hospital initiative. The study indicates that the trend-setting marketing practices embedded in the policies of health care institutions are effective tools in the promotion of BF practices.

The studies in the public health field have spanned along individual (mother), group (demographic) and societal (wider community) levels, apart from Labbok et al (2006) who have discussed the SM link to BF on a more macro-societal level.

### 3.3 Marketing

In both studies allocated within the field of marketing (Mattson and Basu (2010); Lowry, Austin and Patterson (2011)) the primary focus is on the development of SM as a tool where BF practice is the empirical object that illuminates the advancements in tool development.

In the study by Mattson and Basu (2010) the focus is on ‘messaging’ as an important element of SM technique. By messaging the authors refer to the marketing campaign slogans and their meaning to the promoters (marketers) and recipients of the campaigns. While the main advancements are made in terms of marketing theory the empirical case under investigation is an Australian BF campaign designed to change attitudes and increase public awareness about BF. The results of this study suggest that in order to promote BF as a social practice, the consistency of messaging creation, implementation and evaluation, as well as audience analysis in terms of their information needs, is of high importance.

While still placing marketing as a primary focus of the article, Lowry, Austin and Patterson (2011) offer a more balanced view and development of SM in relation to BF than the article by Mattson and Basu (2010). While problematizing the declining rates of BF in the UK, the authors aim to investigate how different SM techniques, specifically the placement, price, product features and promotion (the 4P’s of marketing), influence female choice of BF. Through focus groups as well as longitudinal studies, the researchers concluded that a variation of different SM tools serves as an important incentive for BF practices.

Both articles in the marketing area have been concerned with the group (demographic) or organizational (firm, institution) level.

## 4. Discussion

The review performed within this literature study on SM and BF has shown that the link between the two is both an important theoretical as well as an empirical field. On the one hand the literature shows that the practices and techniques utilized within SM have an influence on BF practices and perceptions; on the other hand it indicates the need for integration between different disciplines trying to contribute to the investigation of this phenomenon.

### 4.1 Models and Level of Analysis

Positioned within the field of psychology and education, studies on the link between SM and BF tend to concentrate on the group level and to a less extend on societal level. They investigate the underlying group behavioural characteristics and the moderating role of SM and its link to BF practices, drawing broader policy implications. In this particular field of studies SM is shown as a pedagogical tool which serves as an agent of change. Studies in the field concentrate primarily on group behaviour/psychological factors and less on the development of SM and BF concepts, thus concentrating on the input variable.

**Figure 1:**
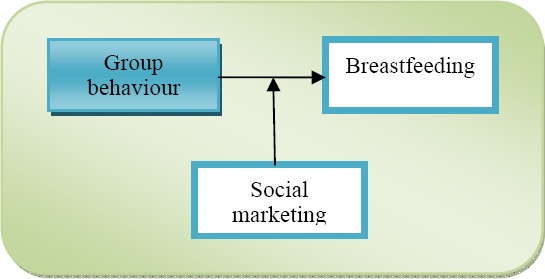
Breastfeeding within the fields of psychology and education

Positioned within the files of public health studies, three levels of analysis aim to contribute to the understanding of the phenomenon: individual (mother), group (demographic group) and societal (wider community) level. The demographic characteristics of both individuals and groups are considered to be important determinants of BF practices. While researchers within this field acknowledge the interrelation between demography and behaviour, the demography in this interrelation is given a major role in studying BF promotion without going deeper into the behavioural specificities. Overall, public health literature puts the primary emphasis on BF as a practice and in its placement within a public health context, while leaving individual/group demographic characteristics as well as SM techniques somewhat in the shade, thus primarily concentrating on the output variable.

**Figure 2:**
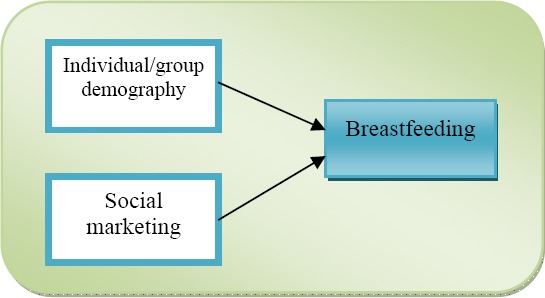
Breastfeeding within the field of public health

Positioned within the field of marketing, the relation between SM and BF spans two levels of analysis: group as well as organizational level (the latter being the outcome of marketing as a discipline belonging to the wider field of organizational/business administration studies). The primary focus in this discipline is on SM as a technique and its development as an effective tool in promoting special practices. While acknowledging the importance of the behaviour of target groups as well as practices to be promoted (BF) the field views them as supplementary to its investigation of SM practice. In line with education and psychology streams, marketing can be seen as a moderating variable in the relation between group behaviour and BF. However the primary focus is on SM techniques rather than targeting social grouping and/or BF practices.

**Figure 3:**
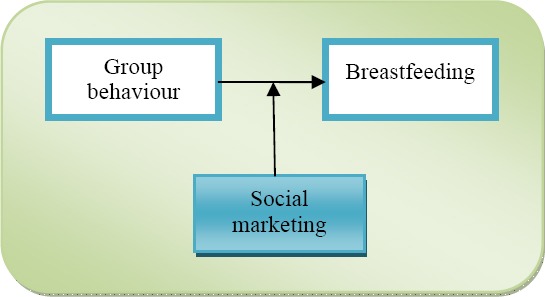
Breastfeeding within the field of marketing

### 4.2 Commonalities and Differences

What emerges from the results and the discussion above is a field of research that while operating with the same concepts of interest, investigates different priorities assigned to them. At the same time as these findings are hardly surprising the literature reviewed indicates that the relationship between the concepts, irrespective of the emphasis, is considered to be of importance in all three broadly defined disciplines. The majority of the studies reviewed here view BF as important practice, and most of the articles while not putting emphasis on BF outcomes for child health, indirectly acknowledge the positive link. Most of the studies reviewed also -implicitly or explicitly- acknowledge that the primary concern with BF is not the lack of education – since most of the mothers are aware of “breast is best”, but the lack of resources such as time, energy, material and social support. When drawing on the connection between BF and SM most of the studies however put more emphasis on SM as a tool of persuasion rather than a tool of education in advocacy for BF. This pattern partly reflects the criticism put forward by Knaak (2006) who argues that BF literature usually discusses BF promotion as a persuasion campaign rather than going deeper into investigating and deconstructing BF promotion as a public health practice.

At the same time the majority of the studies reviewed here are carefully crafted in investigating behavioural patterns and socio-demographics that Knaak has argued are often being put aside in the literature on BF, which shows that a certain field development has taken place since Knaak’s critical publication (2006). As stated previously, the majority of the studies reviewed have concentrated on SM as a tool of persuasion rather than a tool of education, with three studies in the psychology/educational literature reviewed here showing a more weighted conceptualisation of SM. In the aforementioned studies SM is shown as a tool of education, social inclusion as well as health promotion.

Studies in public health reviewed here provide a broader discussion on BF as a practice. They aim to present a view on BF from different levels of analysis thus presenting it as a cross-level phenomenon which in turn allows for a broader and critical conceptualisation of BF. This appears to be lacking in the studies reviewed in the other two disciplines (marketing and psychology/education). Studies reviewed within the field of marketing and public health view SM as a promotional rather than an educational tool. By putting the emphasis on the operational nature of SM as a technique, marketing literature offers a hands-on approach compared to a more diffused discussion on SM provided by public health and psychology/education literature.

### 4.3 Research Gap and New Configuration

**Figure 4:**
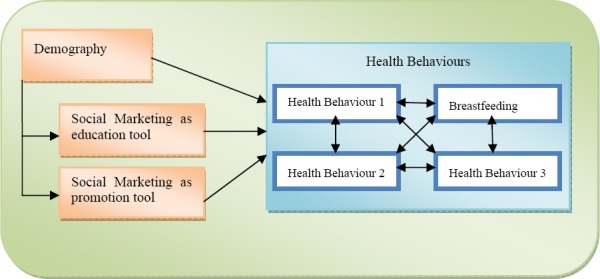
Social marketing-breastfeeding framework

The use of SM in health promotion appears to be an emergent phenomenon that has seen its application on the variety of health related practices (Griffith, Blair-Stevens, & Thorpe, 2008). Identifying BF as one of such practices this article has set as its aim (1) to identify the research gaps that exist between different disciplines within which SM and BF are being investigated as well as (2) to suggest a theoretical framework which could be used in future empirical studies on the subject. Through the literature review this article has established that the relationship between SM and BF has been divided between three broadly identified disciplines, namely psychology/education, public health and marketing which have used different ways of modelling the relationship as well as putting specific emphasis on either SM, BF, or socio-demographic and/or behavioural factors. Moreover by analysing the differences this article has identified potential strengths and weaknesses of the research in those different fields. Drawing on these findings a new configuration is put forward that attempts to illuminate the strengths and eliminate possible weaknesses identified through the review (Figure 4.)

Borrowing from psychology/education literature on the relationship between SM and BF, it is suggested that SM should be considered from two angles: SM as a promotional and SM as an educational tool. In doing so one would be able to better understand how the use of these two different strategies could be (1) combined with each other to be further used as an effective tool of driving health-related outcome, as well as (2) to understand how positioning of SM from an educational and promotional perspective might influence these outcomes in a different manner. It is also argued that defining SM as a construct within these two strategies require understanding of SM techniques. It is here that marketing literature could contribute with refined definitions of techniques as well as suggestions of how effectiveness of these techniques can be used in targeting specific segments. This puts further emphasis on considering the socio-demographic variables both as a way to understanding the functioning of SM and its effectiveness in conjunction with demographic characteristics as well as the explanatory factor of health behaviour, of which BF is a part. It is also proposed that instead of considering BF as an isolated health behaviour one should put it into a broader context with other health behaviours; a notion found to be advocated within the public health literature reviewed. By doing so BF could be investigated in relation to other health behaviours including, but not limited to, physical activities, drinking and smoking, mental and sexual health. Finally, one should also consider the environmental influences such as economic and social conditions, as well as national and local specificities within which the relation between SM and BF is being investigated. It might be reasonable to assume that depending e.g. on the human development of the society as well as technological advances within a given community or nations, the influences of SM and socio-demographic factors on BF as a part of health behaviour could differ. While the model might appear to be complex, the linkages of the new framework advocated here have been established and investigated previously albeit within different disciplines of research, the complexity thus lies not in the model itself but in the ability to draw from the strengths of each discipline while avoiding its pitfalls.

### 4.4 Future Research and Limitation

Based on the discussion above it is suggested that further different venues for inquiry into the link between SM and BF could be taken up. First, it is posed that the concept of SM within health sciences might need further refinement, especially in developing the idea of SM as being not only a promotional but also an educational tool. Second, future studies could inquire into the interplay of SM practices with socio-demographic factors in their influence on health behaviour. By considering this interplay, research might find the combination of SM techniques and demographic factors that target specific health behaviour. Third, the development of BF as a concept within a wider context of health behaviour could be yet another venue for future research. Uncovering the interrelations between different health behaviours and BF could help the researchers and practitioners alike to understand BF practices. Fourth, exploring the relationship between SM and BF in different contexts could also be of interest for a future research theme. It appears that studies usually concentrate on one particular context which in turn might be a strong determinant of the relationship between SM and BF. In order to develop better understanding and to create a general theory it is thus argued that researchers should aim at performing cross-context studies studying for example both developed and developing countries.

This study is however not without its limitations. In this study a general terminology of ‘social marketing’ and ‘breastfeeding’ has been used in an attempt to investigate the relationship between the two concepts. That has been decided under the assumption that the key word ‘social marketing’ covers the application of marketing in social issues as well as being used with researchers’ awareness of its difference from advertising or promotional campaigns. This however could lead to the exclusion of some relevant papers while using the terminology of promotion campaigns as a connotation for SM practices. When it comes to the word breastfeeding (or breast-feeding or breast feeding), one could not exclude the possibility that other terms such as suckle or lactate (lactation) could have been used by other researchers to refer to the practice, however it has been assumed that BF could be deemed as a more universally used term in scientific literature to describe the practice of human lactation.
